# Supplementary material for: Quantum vertex model for reversible classical computing
Source: Nat Commun. 2017 May 12;8:15303. doi: 10.1038/ncomms15303 (PMC5437300; doi:10.1038/ncomms15303)
Supplement: Supplementary Information — Supplementary Table and Supplementary Notes. [file ncomms15303-s1.pdf]

Supplementary Information
Quantum Vertex Model for Reversible Classical Computing

Supplementary notes

Supplementary note 1: Building TOFFOLI gates with one-and two-body interactions

In this section, we present the table with all possible configurations of a TOFFOLI gate formulated in a rectangular tile, and their corresponding energies given by

$$\begin{aligned} E_{\text{TOFFOLI}}(\sigma_a, \sigma_b, \sigma_c; \sigma_{a'}, \sigma_{b'}, \sigma_d; \sigma_S) &= -J(\sigma_a\sigma_{a'} + \sigma_b\sigma_{b'}) + J(\sigma_a - 3\sigma_b - 2\sigma_c + 2\sigma_d + 4\sigma_S) \\ &\quad + J(-3\sigma_a\sigma_b - 2\sigma_a\sigma_c + 4\sigma_b\sigma_c + 2\sigma_a\sigma_d - 4\sigma_b\sigma_d - 4\sigma_c\sigma_d \\ &\qquad + 4\sigma_a\sigma_S - 8\sigma_b\sigma_S - 6\sigma_c\sigma_S + 6\sigma_d\sigma_S). \end{aligned} \quad (1)$$

From which it is clear that the states which satisfy the gate constraint all stay in the ground state manifold of the Hamiltonian.

The TOFFOLI gate takes a three-bit input sate (a, b, c) into $(a, b, ab \oplus c)$. The copying of the first two bits is trivially enforced by a ferromagnetic coupling: $-J(\sigma_a\sigma_{a'} + \sigma_b\sigma_{b'})$, so we shall only list the part that enforces the third output bit: $d = ab \oplus c$ in the table below. As explained in Methods, in order to achieve that with at most two-body interactions, one needs an ancilla bit that we call S.

In Supplementary Table 1, the first eight states separated by double lines span the ground state manifold of Hamiltonian (1), and one can readily check that they indeed satisfy the gate constraint imposed by the TOFFOLI gate. All other unsatisfying states have energy scales higher by multiple of J. Therefore in the limit where J is much larger than any other energy scale in the system (K, Γ and T), one essentially projects out the ground state manifold. Notice that there are cases when (a, b, c, d) satisfies the gate constraint, but the ground state only picks one value of S. For example, the state $(a, b, c, d) = (0, 0, 1, 1)$ satisfies the gate constraint, but the ground state manifold only picks $S = 0$, and $S = 1$ has a higher energy. This does not cause a problem because the ancilla bit does not enter the computation, and its value does not really matter.

a	b	c	d	S	σ_a	σ_b	σ_c	σ_d	σ_S	E
0	0	0	0	0	$-$	$-$	$-$	$-$	$-$	$-13J$
0	0	1	1	0	$-$	$-$	$+$	$+$	$-$	$-13J$
0	1	0	0	1	$-$	$+$	$-$	$-$	$+$	$-13J$
0	1	1	1	1	$-$	$+$	$+$	$+$	$+$	$-13J$
1	0	0	0	0	$+$	$-$	$-$	$-$	$-$	$-13J$
1	0	1	1	0	$+$	$-$	$+$	$+$	$-$	$-13J$
1	1	0	1	0	$+$	$+$	$-$	$+$	$-$	$-13J$
1	1	1	0	1	$+$	$+$	$+$	$-$	$+$	$-13J$
0	0	0	1	0	$-$	$-$	$-$	$+$	$-$	$-9J$
0	0	1	0	1	$-$	$-$	$+$	$-$	$+$	$-9J$
0	1	0	1	0	$-$	$+$	$-$	$+$	$-$	$-9J$
0	1	1	0	1	$-$	$+$	$+$	$-$	$+$	$-9J$
1	0	1	0	0	$+$	$-$	$+$	$-$	$-$	$-9J$
1	1	0	0	0	$+$	$+$	$-$	$-$	$-$	$-9J$
1	1	0	0	1	$+$	$+$	$-$	$-$	$+$	$-9J$
1	1	1	1	0	$+$	$+$	$+$	$+$	$-$	$-9J$
1	1	1	1	1	$+$	$+$	$+$	$+$	$+$	$-9J$
0	0	1	0	0	$-$	$-$	$+$	$-$	$-$	$-J$
0	1	0	1	1	$-$	$+$	$-$	$+$	$+$	$-J$
1	0	0	1	0	$+$	$-$	$-$	$+$	$-$	$-J$
1	0	1	0	1	$+$	$-$	$+$	$-$	$+$	$-J$
0	0	0	0	1	$-$	$-$	$-$	$-$	$+$	$3J$
0	0	1	1	1	$-$	$-$	$+$	$+$	$+$	$3J$
0	1	0	0	0	$-$	$+$	$-$	$-$	$-$	$3J$
0	1	1	1	0	$-$	$+$	$+$	$+$	$-$	$3J$
1	1	0	1	1	$+$	$+$	$-$	$+$	$+$	$11J$
1	1	1	0	0	$+$	$+$	$+$	$-$	$-$	$11J$
1	0	0	0	1	$+$	$-$	$-$	$-$	$+$	$19J$
1	0	1	1	1	$+$	$-$	$+$	$+$	$+$	$19J$
0	0	0	1	1	$-$	$-$	$-$	$+$	$+$	$31J$
0	1	1	0	0	$-$	$+$	$+$	$-$	$-$	$31J$
1	0	0	1	1	$+$	$-$	$-$	$+$	$+$	$55J$

Supplementary Table 1: All possible configurations of a TOFFOLI gate formulated in a rectangular tile with an ancilla S, and their corresponding energies given by Eq. (1). The first eight states span the ground state manifold, and they satisfy the gate constraint.

Supplementary note 2: Nearest neighbor vertex couplings

The $K^{g_s g_{s'}}_{q_s,q_{s'}}$ couplings encode the energy cost for mismatched nearest-neighbor vertices. Here we give an example of how these matrix elements are constructed. Consider two adjacent vertices at s and s' that enforce the TOFFOLI gate: $g_s = g_{s'}$ =TOFFOLI (or T for short). The matrix elements of $K^{T,T}_{q_s,q_{s'}}$ basically count the number of bits that are mismatched between the output state $G(q_s)$ of one gate, where G is the gate function, and the input state $q_{s'}$ of the other gate, for $q_{s,s'} = 0, 1, \dots, 7$.

When the two sites s and s' share a single bond, the 8×8 matrix $K^{T,T}_{q_s,q_{s'}}$ (with $q_{s,s'} = 0, 1, \dots, 7$)

is given by

$$K^{T,T} = \begin{bmatrix} 0 & K & 0 & K & 0 & K & 0 & K \\ 0 & K & 0 & K & 0 & K & 0 & K \\ 0 & K & 0 & K & 0 & K & 0 & K \\ 0 & K & 0 & K & 0 & K & 0 & K \\ K & 0 & K & 0 & K & 0 & K & 0 \\ K & 0 & K & 0 & K & 0 & K & 0 \\ K & 0 & K & 0 & K & 0 & K & 0 \\ K & 0 & K & 0 & K & 0 & K & 0 \end{bmatrix}, \quad (2)$$

and when the sites share a double bond, the 8×8 matrix is given by

$$K^{T,T} = \begin{bmatrix} 0 & 0 & K & K & K & K & 2K & 2K \\ K & K & 0 & 0 & 2K & 2K & K & K \\ K & K & 2K & 2K & 0 & 0 & K & K \\ 2K & 2K & K & K & K & K & 0 & 0 \\ 0 & 0 & K & K & K & K & 2K & 2K \\ K & K & 0 & 0 & 2K & 2K & K & K \\ 2K & 2K & K & K & K & K & 0 & 0 \\ K & K & 2K & 2K & 0 & 0 & K & K \end{bmatrix}, \quad (3)$$

where K is the ferromagnetic energy scale that penalizes configurations in which the states of adjacent vertices are incompatible.

Supplementary note 3: Bounds on number of replicas needed for learning algorithm

Below we estimate the number of replicas, N_R required to achieve the correct assignment of gates in the "annealing with learning" algorithm described in Results with accuracy ϵ. Assume that the probability of correct assignment of a gate within each replica is p. The probability that k of the N_R replicas assign the wrong identity to a given gate is given by the binomial distribution:

$$P(N_R, k; p) = \frac{N_R!}{(N_R - k)!k!} p^{N_R - k}(1-p)^k. \quad (4)$$

The probability that a fraction greater than α of the N_R replicas ($N_R \gg 1$) assign the wrong identity to a particular gate can then be written as:

$$\begin{aligned} P_w(N_R, \alpha; p) &= \sum_{k=\alpha N_R}^{N_R} \frac{N_R!}{(N_R-k)!k!} p^{N_R-k}(1-p)^k \\ &< \frac{N_R!}{(\frac{N_R}{2}!)^2} \sum_{k=\alpha N_R}^{N_R} p^{N_R-k}(1-p)^k \quad (5) \\ &\approx \frac{[2p^{1-\alpha}(1-p)^\alpha]^{N_R}}{\frac{(2p-1)}{p}}. \end{aligned}$$

It then follows that, for fixed $p > 1/2$, N_R and α, the probability that a correct assignment is made to all LW gates by a fraction of the replicas greater than α is $1 - \epsilon$ is given by:

$$\begin{aligned} P_c(N_R, \alpha; p) &= [1 - P_w(N_R, \alpha; p)]^{LW} \\ &= 1 - \epsilon \quad (6) \\ &> 1 - LW \, \frac{[2p^{1-\alpha}(1-p)^\alpha]^{N_R}}{\frac{(2p-1)}{p}}, \end{aligned}$$

which implies that the number of replicas, $N_{R\epsilon}$, needed to ensure an error rate smaller than ϵ is given by:

$$N_{R\epsilon} = \frac{\ln\left[\frac{2p-1}{p}\frac{\epsilon}{LW}\right]}{\ln\left[2p^{1-\alpha}(1-p)^{\alpha}\right]}. \tag{7}$$

We note that the above argument assumes that the states of the gates are uncorrelated; for a given N_R the correlations built into the vertex model should lead to a lower error rate than the estimate given here.
